# Metformin Protects against Oxidative Stress Injury Induced by Ischemia/Reperfusion via Regulation of the lncRNA-H19/miR-148a-3p/Rock2 Axis

**DOI:** 10.1155/2019/8768327

**Published:** 2019-12-16

**Authors:** Jing Zeng, Long Zhu, Jing Liu, Tao Zhu, Zhaohui Xie, Xiaoou Sun, Hao Zhang

**Affiliations:** ^1^School of Basic Medicine, Xiang Nan University, Chenzhou 423000, China; ^2^HEC Research and Development Center, HEC Pharm Group, Dongguan 523871, China; ^3^School of Life Science and Bioengineering, Henan University of Urban Construction, Pingdingshan 467000, China; ^4^Institute of Biomedical and Pharmaceutical Sciences, Guangdong University of Technology, Guangzhou 510006, China

## Abstract

Previous studies have shown that metformin not only is a hypoglycemic agent but also has neuroprotective effects. However, the mechanism of action of metformin in ischemic stroke is unclear. Oxidative stress is an important factor in the pathogenesis of cerebral ischemia-reperfusion injury. It has been reported that metformin is associated with stroke risk in the clinical population. This study is aimed at investigating the effect and mechanism of metformin in an experimental model of oxidative stress induced by ischemia/reperfusion (I/R) *in vivo* and oxygen glucose deprivation/reperfusion (OGD/R) *in vitro*. Metformin (100, 200, and 300 mg/kg) was administered intraperitoneally immediately after induction of cerebral ischemia. The indicators of oxidative stress selected were antioxidant enzyme activities of catalase, malondialdehyde (MDA), nitric oxide (NO), superoxide dismutase (SOD), and glutathione peroxidation enzyme (GSHPx). First, we demonstrated that metformin can significantly alleviate acute and chronic cerebral I/R injury and it has a strong regulatory effect on stroke-induced oxidative stress. It can reduce the elevated activities of MDA and NO and increase the levels of GSHPx and SOD in the cerebrum of mice and N2a cells exposed to I/R. Furthermore, real-time PCR and western blot were used to detect the expression of long noncoding RNA H19 (lncRNA-H19), microRNA-148a-3p (miR-148a-3p), and Rho-associated protein kinase 2 (Rock2). The direct interaction of lncRNA-H19, miR-148a-3p, and Rock2 was tested using a dual luciferase reporter assay. lncRNA-H19 altered OGD/R-induced oxidative stress by modulating miR-148a-3p to increase Rock2 expression. The expression of lncRNA-H19 and Rock2 could be downregulated with metformin *in vivo* and *in vitro*. In conclusion, our study confirmed that metformin exerts neuroprotective effects by regulating ischemic stroke-induced oxidative stress injury via the lncRNA-H19/miR-148a-3p/Rock2 axis. These results provide new evidence that metformin may represent a potential treatment for stroke-related brain injury.

## 1. Introduction

Stroke is a serious and common disease that is one of the main causes of disability and death worldwide [1, 2]. It is increasingly understood that an excessive inflammatory response and oxidative stress are closely related to the pathogenesis of cerebral ischemia-reperfusion (I/R) injury [3]. One of the early and most important components of cerebral I/R is reactive oxygen species- (ROS-) induced tissue damage [4]. Due to the complex structure of the brain, its relatively low antioxidant capacity, its high oxidative metabolism activity, insufficient cell repair activity of neurons, and high polyunsaturated fatty acid (PUFA) content, the brain is extremely susceptible to oxidative stress-induced damage [5]. Excessive production of ROS leads to oxidative injury, including DNA damage, protein oxidation, and lipid peroxidation, which can lead to cell apoptosis or necrosis [6].

Long-chain noncoding RNAs (lncRNAs) are a type of noncoding RNA containing more than 200 bases that regulate the transcription and translation of genes, epigenetic mechanisms, cell differentiation, and other physiological/pathological activities [7]. Many studies have shown that lncRNAs also affect many physiological and pathological processes of the nervous system and they have been determined as potential biomarkers for stroke [8]. For example, there is evidence that elevated expression of lncRNA-H19 is closely related to the progression of cerebral ischemia [9]. lncRNA-H19 is a maternally derived gene localized to human chromosome 11 that has been confirmed to be associated with the susceptibility of the Chinese population to stroke [10]. lncRNA-H19 not only protects H9c2 cells from hypoxia-induced damage by regulating miR-139 but also acts as a positive regulator of autophagy in cerebral ischemia [11]. Interestingly, the function of lncRNA is usually mediated through the regulation of microRNAs that regulate mRNA expression by binding to the 3′ untranslated region (3′UTR) after transcription [12]. For example, lncRNA-H19 negatively modifies cell differentiation by direct targeting of miR-29b-3p followed by inhibition of TGF-*β*1 COL1A1 expression [13]. Based on miRNA expression profiling in ischemic stroke, it has been shown that some miRNAs may be potential biomarkers for the diagnosis of stroke or prediction of prognosis.

It has been previously reported that miR-148a can alleviate hepatic I/R injury in mice via modulating Ca^2+^/calmodulin-dependent protein kinase II alpha (CaMKIIalpha) [14]. New evidence also suggests that miR-148a can regulate microglial inflammation induced by hypoxia glucose through the MAPK pathway, which is crucial in I/R-induced injury [15]. Many reports have confirmed that miR-148a-3p is processed from the precursor of miR-148a, so it has a similar activity to miR-148a [16]. We speculated that miR-148a-3p also plays a very important role in cerebral I/R injury. Through bioinformatic analysis, the combination of miR-148a-3p and Rock2 was proposed to target and regulate the expression of Rock2. Interestingly, Rock2 can mediate heme oxygenase-1/nuclear factor erythroid-2-related factor 2 (HO-1/Nrf2) to regulate oxidative stress injury induced by hypoxia, and oxidative stress injury is a crucial factor in the pathogenesis of cerebral ischemic stroke [17]. The HO-1/Nrf2 molecular axis has been reported to participate in the oxidative stress response, including that induced by cerebral ischemia [18, 19]. The mechanism of action of this process remains unclear. The purpose of this research is to explore whether lncRNA-H19 promotes HO-1/Nrf2 signaling by acting on downstream miR-148-3p and Rock2 to mediate oxidative stress induced by cerebral I/R. It is speculated that lncRNA-H19 may act on miR-148a-3p to regulate the existence and aggravation of stroke.

Metformin is used to treat type 2 diabetes [20]. Its role is mainly to reduce the glucose output of the liver by inhibiting gluconeogenesis. New research confirms chronic metformin treatment reduces the risk of stroke and reduces cardiovascular mortality by 26% in the clinical population [21]. This protective effect is not linked to its hypoglycemic effect. Many studies have shown that metformin has antioxidant properties and can participate in cardiovascular protection [22]. The antioxidant properties of metformin may clarify the new pharmacological transduction pathways of this medicine via the regulation of redox dependence. Studies have shown that metformin can improve cognitive ability in a mouse model of Alzheimer's disease [23, 24]. These results indicate that metformin may have a protective effect in neurological diseases caused by neuronal damage. Although metformin has a protective effect against neuronal injury induced by cerebral I/R, its mechanism of action is still unclear.

In the current study, we investigated the effect of metformin on oxidative stress using *in vitro* and *in vivo* ischemic stroke models. In addition, we found that lncRNA-H19, a molecular sponge, competitively binds to miR-148a-3p and is regulated by metformin. Metformin may represent a potential therapeutic agent for the treatment of ischemic stroke. Our study illustrates that metformin prevents brain ischemia by accommodating I/R-induced oxidative stress via modulation of the lncRNA-H19/miR-148a-3p/Rock2 axis.

## 2. Materials and Methods

### 2.1. Animals and Ischemic Model

Adult male C57BL/6 mice (9–13 weeks, 21–24 g) were obtained from the Animal Research Centre of Guangzhou University of Chinese Medicine (Guangzhou, China). The mice were placed in a temperature-adjustable environment (22°C ± 1°C) with six per cage on a 12 h light/12 h dark cycle with free access to food and water. Animals were randomly divided into sham control and experimental groups. Our research was authorized by the Institutional Animal Care and Use Committee of Guangdong Pharmaceutical University.

We simulated transient cerebral ischemia using the previously described middle cerebral artery occlusion (MCAO) model [25–27]. Operated mice were subjected to 1.5 h of ischemia before 24 h reperfusion. The sham group underwent the same surgery except for the MCAO filament inducement.

### 2.2. Metformin Administration

Metformin purchased from Sigma (St. Louis, MO, USA) was dissolved in sterile saline, and different doses (100, 200, and 300 mg/kg) were administered by intraperitoneal injection immediately after ischemia and then given once a day until the mice were euthanized. The control group was injected with an equal amount of physiological saline.

### 2.3. Evaluation of Neurological Deficits and Cerebral Infarct Volume

After 24 h of MCAO, mice were evaluated for neurological dysfunction based on the modified neurological severity score (mNSS). The detailed scoring system of mNSS is described in previous literature [28]. The mice were evaluated for neurological deficits and sacrificed by intraperitoneal injection of 400 mg/kg chloral hydrate. With the help of the brain matrix, the brain was removed and continuously cut into 5 slices that were 1 mm thick. We used 1.5% 2,3,5-triphenyltetrazolium chloride (TTC) staining to determine the cerebral infarction as described above [29].

### 2.4. Measurement of Edema

After 24 h of MCAO, cerebral edema was detected by comparing the weight ratio of wet tissue to dry tissue, as previously described [30].

### 2.5. HE Staining and Immunohistochemistry Assays

After 24 h of MCAO, brain tissue dissection was performed for histopathological analysis (*n* = 3). We used hematoxylin and eosin (HE) or immunohistochemical staining to measure the degree of damage caused by the cerebral infarction, as previously described [31].

### 2.6. TUNEL (Terminal Deoxynucleotidyl Transferase dUTP Nick End Labeling) Assays

Neuronal apoptosis was detected by TUNEL assay. The cells or brain tissue fraction was fixed on the slide with ice-cold 4% paraformaldehyde for 20 min followed by permeabilization with 0.1% (*v*/*v*) Triton X-100 (Sigma) for 5 min. The slides were incubated with the TUNEL incubation mixture for 1.5 h at 37°C in a wet and dark environment. The nuclei were then stained with 4,6-diphenylamine-2′-phenylhydrazine hydrochloride (DAPI). Positive TUNEL staining was observed with a fluorescence microscope and photographed (Leica DM4000 bleeding, Wetzlar, Germany). The apoptotic index is the percentage of TUNEL-positive cells (positive cells/100% total cells).

### 2.7. *In Vivo* lncRNA-H19 Overexpression

The pcDNA3.1-H19 was purchased from RiboBio (Guangzhou, China), and the negative control was a pcDN3.1-NC. The mice were placed in a stereotactic apparatus and deeply anesthetized with 1.5%–3% isoflurane. We injected 3 *μ*L plasmid into the right ventricular cannula of the mouse brain at a rate of 0.2 *μ*L/min, as previously described [32]. The cannula was maintained for 3 min before extubation. Two days after lentiviral transfection, the mice underwent MCAO.

### 2.8. Determination of MDA, SOD, and GSHPx Levels

The levels of SOD, GSHPx, and MDA in N2a cells and mouse brain tissues were detected using a kit purchased from Beyotime (Jiangsu, China).

### 2.9. N2a Cell Culture and Oxygen Glucose Deprivation (OGD) Treatment

The N2a cells used in the experiments were obtained from the American Type Culture Collection (ATCC, Manassas, VA, USA). N2a cells were cultured in Dulbecco's Modified Eagle's Medium (DMEM) supplemented with 100 U/mL penicillin, 100 *μ*g/mL streptomycin, and 5% (*v*/*v*) heat-inactivated fetal bovine serum (FBS). The cells were cultured at 37°C in a humidified environment containing 5% CO_2_, and the medium was updated every 1.5 days. The cells were cultured for an additional 24 h in a humidified incubator. The cells in the OGD group were incubated in a simulated ischemic solution and stored at 37°C for 4 h in an anoxic incubator filled with 95% N_2_/5% CO_2_. The cells were transferred to normal medium for 24 h and incubated at 37°C in an incubator containing 5% CO_2_, and reperfusion occurred.

### 2.10. Nitric Oxide (NO) and Lactate Dehydrogenase (LDH) Assay

The cytotoxicity of ODG/R in N2a cells was determined based on the release of NO and LDH into the incubation medium [33]. The assays were performed in accordance with the manufacturer's instructions.

### 2.11. Measurement of Mitochondrial Transmembrane Potential (MMP)

We used the fluorescent dye JC-1 to determine MMP. First, the cultured cells were treated with OGD/R and metformin, then incubated with JC-1 staining solution (5 *μ*g/mL) for 15 min at 37°C. Next, the cells stained with JC-1 were washed three times in buffer. The fluorescence intensity detected by the total amount of JC-1 was 525 nm and the emission wavelength was 590 nm. The JC-1 monomer was measured using a microplate reader at an excitation wavelength of 490 nm and an emission wavelength of 530 nm. The ratio of the fluorescence intensity of the polymer to the monomer was examined as a standard for MMP.

### 2.12. Determination of Intracellular ROS Content

Intracellular ROS content was detected as described previously [34].

### 2.13. Cell Transfection

Regarding the interference of H19, we transfected lncRNA-H19 siRNA (siRNA-H19) or control siRNA into N2a cells. In order to knock down the level of miR-148a-3p, cells were transfected with miR-148a-3p inhibitor and a negative control (NC inhibitor) was used as a control. To overexpress lncRNA-H19, we transfected the recombinant pcDNA3.1 plasmid of the lncRNA-H19 gene into N2a cells with a pcDNA3.1-NC empty plasmid as control. Regarding miR-148a-3p overexpression, cells were transfected with miR-148a-3p mimics and a negative control (NC mimic). All transfections were performed using lipofectamine 2000 according to the manufacturer's guidelines, and cell samples were collected 48 h later for subsequent experimental studies.

### 2.14. Luciferase Reporter Assay

The binding site of lncRNA-H19 to miR-148a-3p was analyzed using the bioinformatics website http://starbase.sysu.edu.cn. The lncRNA-H19 3′UTR fragment containing the predicted miR-148a-3p binding site was cloned into the pmirGLO vector (RiboBio, Guangzhou, China) to form the reporter vector H19-wild-type (H19-WT). Next, we generated the lncRNA-H19 mutant (H19-MUT) by site mutation to mutate the putative binding site of miR-148a-3p in the lncRNA-H19 3′UTR. The miR-148a-3p mimic or the miRNAs and the vector (H19-WT or H19-MUT) were cotransfected into N2a cells, and luciferase activity was detected using a dual luciferase reporter assay system. The miR-148a-3p and Rock2 luciferase reporter assays were identical to those described above.

### 2.15. Real-Time Polymerase Chain Reaction (RT-PCR)

Total RNA was extracted from the N2a cell line or C57BL/6 mouse cortex using the RNaEXTM Total RNA Isolation Kit (Generay, Shanghai, China) according to the manufacturer's instructions. RT-PCR amplification was performed using ABI7500, and RT-PCR detection was performed using mRNA and miRNA qPCR detection kits (CWBIO, Beijing, China). Detailed steps and methods of operation for RT-PCR experiments can be found in previous studies [35, 36]. The RT-PCR primer sequence was synthesized by Generay (oligo sequences are shown in Table 1).

### 2.16. Western Blot Analysis

Brain tissues and N2a cells were lysed using low-temperature RIPA buffer (CWBIO, Beijing). 10 *μ*g of total protein was resolved on 8–15% sodium dodecyl sulfate-polyacrylamide gel electrophoresis (SDS-PAGE) gels and transferred to polyvinylidene (PVDF) membranes. Membranes were probed with the primary antibodies including anti-Rock2 (9029; Cell Signaling Technology), anti-endothelial nitric oxide synthase (eNOS; 32027; Cell Signaling Technology), anti-HO-1 (43966; Cell Signaling Technology), anti-Nrf2 (ab89443; Abcam), anti-glyceraldehyde 3-phosphate dehydrogenase (GAPDH; ab181602, Abcam) antibodies. The membrane was then incubated with horseradish peroxidase-conjugated secondary antibodies, and protein signals were observed with ECL reagents.

### 2.17. Statistical Analysis

Data are represented as means ± standard deviation (SD). Data analysis was performed using one-way analysis of variance (ANOVA) followed by post hoc analysis (Tukey's multiple comparison test) using SPSS 20.0 software (IBM, Armonk, NY, USA). All experiments were repeated at least three times. Differences with a *p* < 0.05 were defined as being statistically significant.

## 3. Results

### 3.1. Metformin Protects against Brain Damage following MCAO/R

**Figure 1:**
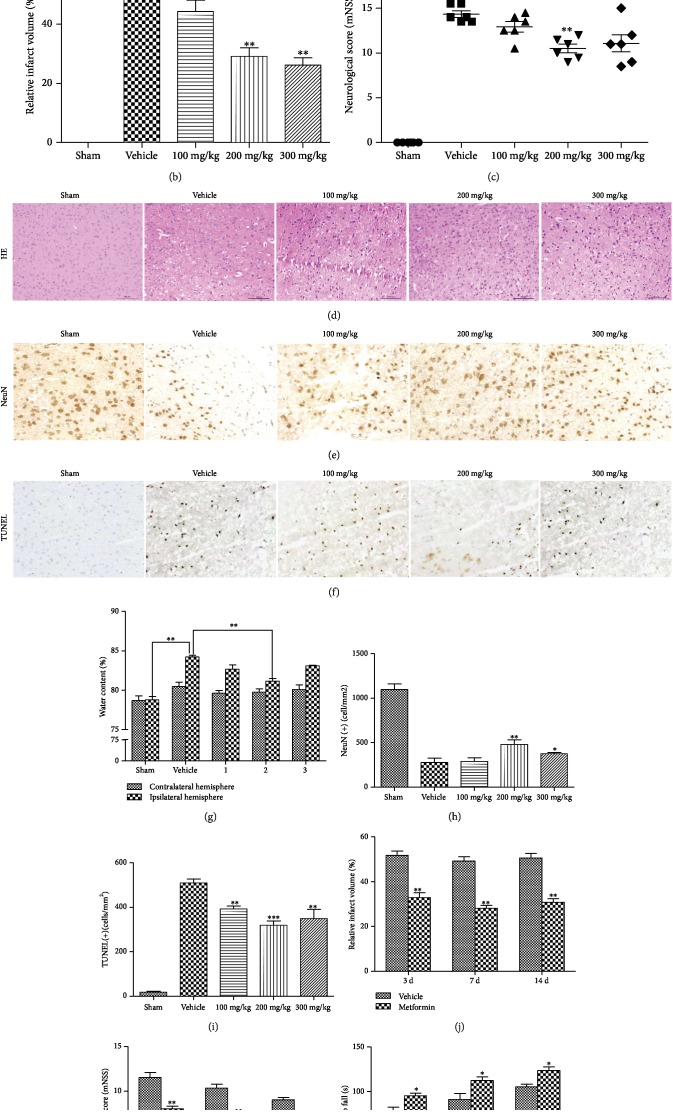
Effects of metformin on various parameters measured 24 hours after tMCAO in mice. (a) Infarction in serial brain sections stained by TTC (magenta: healthy tissue; white: damaged tissue). (b) Statistical analysis of the percentage of infarct volume was determined for each group. (c) Neurological scores after transient middle cerebral artery occlusion (tMCAO) in the vehicle and metformin treatment groups. (d) Hematoxylin eosin stains of coronal sections of the brain after 24 hours of reperfusion in the dose-response study groups (*n* = 3 per group). Scale bar = 100 *μ*m. (e) Effects of metformin on neuronal immunoreactivity in tMCAO mice (*n* = 3 per group). Scale bar = 100 *μ*m. (f) Effect of metformin on apoptosis in the mice ischemic brain (*n* = 3 per group). Scale bar = 100 *μ*m. (g) Hemispheric water content. Water content in the ischemic and nonischemic contralateral brain hemispheres studied 24 hours after 1.5 hours of MCAO in mice with and without metformin administration. Histograms represent the mean ± SD (*n* = 6). ^∗∗^*p* < 0.01 versus the vehicle group by 1-way analysis of variance with Tukey's multiple comparison test. (e) Number of NeuN-immunopositive cells/mm^2^ of brain section. Histograms represent the mean ± SD. ^∗^*p* < 0.05 versus the vehicle group by 1-way analysis of variance with Tukey's multiple comparison test. (f) Number of TUNEL-positive cells/mm^2^ in the brain sections. Data were expressed as the mean ± SD (*n* = 3 per group). ^∗^*p* < 0.05 and ^∗∗^*p* < 0.01 versus the vehicle group; ^#^*p* < 0.05 versus the edaravone group by 1-way analysis of variance with Tukey's multiple comparison test. (j) Statistical analysis of the percentage of infarct volume was determined for each group at different reperfusion time points (3, 7, and 14 d). Data were expressed as the mean ± SD (*n* = 3 per group). ^∗^*p* < 0.05 and ^∗∗^*p* < 0.01 versus the vehicle group by 1-way analysis of variance with Tukey's multiple comparison test. (k) Neurological scores after tMCAO in the vehicle and metformin treatment groups at different reperfusion time points (3, 7, and 14 d). Data were expressed as the mean ± SD (*n* = 3 per group). ^∗^*p* < 0.05 and ^∗∗^*p* < 0.01 versus the vehicle group by 1-way analysis of variance with Tukey's multiple comparison test. (l) Latency to fall after tMCAO in the vehicle and metformin treatment groups at different reperfusion time points (3, 7, and 14 d). Data were expressed as the mean ± SD (*n* = 3 per group). ^∗^*p* < 0.05 and ^∗∗^*p* < 0.01 versus the vehicle group by 1-way analysis of variance with Tukey's multiple comparison test.

To investigate whether metformin has neuroprotective effects on cerebral ischemic injury, we calculated the improvement in neurobehavioral function, infarct volume, and brain water content in mouse brain 24 h after MCAO. A significant reduction in infarct size was observed at doses of 200 and 300 mg/kg metformin compared with the vehicle group (Figures 1(a) and 1(b)). No significant neurobehavioral impairment or differences in brain water content were found in the sham group, while a more serious lack of neurobehavioral function and increased brain water content were shown in the MCAO/R group. Treatment with 200 mg/kg metformin markedly improved the situation compared with that in the MCAO/R group (Figures 1(c) and 1(g)). Subsequently, we examined the improvement histologically using HE staining, anti-NeuN immunohistochemistry, and TUNEL assays. The results showed that 200 mg/kg metformin had a protective effect against tissue injury and apoptosis and increased neuronal survival (Figures 1(d)–1(f), 1(h), and 1(i)).

Next, 200 mg/kg metformin was intraperitoneally injected immediately after MCAO, and reperfusion was performed for 3, 7, and 14 days after surgery. As shown in Figures 1(j)–1(l), metformin also had a certain degree of neuroprotective effect on days 3, 7, and 14 after ischemic stroke. These data show that metformin can improve neurological function and infarct volume at both acute and chronic time points after stroke. Therefore, these results provide a basis for the neuroprotective effects of metformin on cerebral ischemic injury in mice. Since 200 mg/kg metformin has the best therapeutic effect, it was the dose used for subsequent experiments.

### 3.2. Metformin Treatment Decreased Oxidative Stress following MCAO/R *In Vivo*

**Figure 2:**
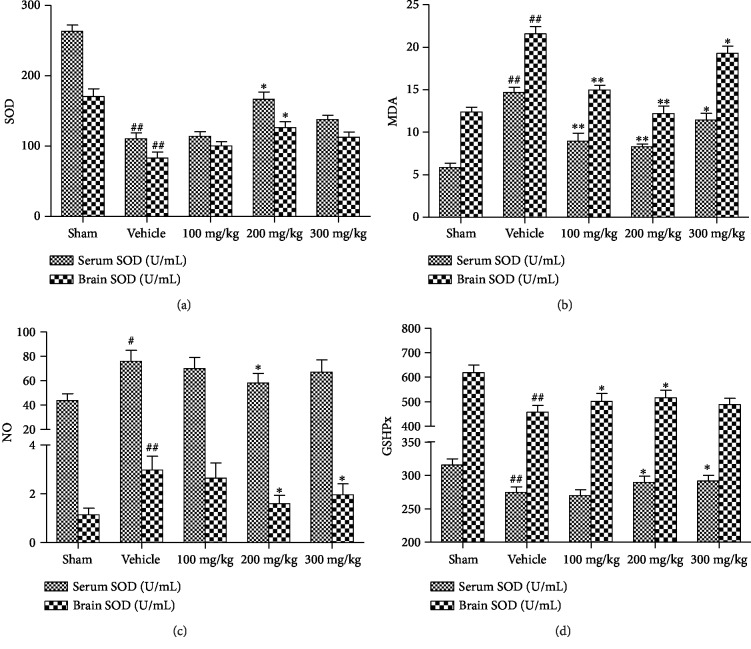
Effect of metformin on the levels of oxidative stress. (a) Activities of SOD in the serum and the brain. (b) Levels of MDA in the serum and the brain. (c) Levels of NO in the serum and the brain. (d) Activities of GSHPx in the serum and the brain. Data were expressed as the mean ± SD (*n* = 5 per group). ^∗^*p* < 0.05 and ^∗∗^*p* < 0.01 versus the vehicle group by 1-way analysis of variance with Tukey's multiple comparison test. ^#^*p* < 0.05 and ^##^*p* < 0.01 versus the sham group by 1-way analysis of variance with Tukey's multiple comparison test.

To understand the effects of metformin on oxidative stress, we investigated the effects of metformin on the levels of SOD, MDA, NO, and GSHPx in serum and brain tissue of mice after MCAO/R. The levels of MDA and NO in the MCAO/R group were significantly higher than those in the sham operation group. Metformin reduced MDA and NO levels compared to the MCAO/R group (Figures 2(b) and 2(c)). Meanwhile, metformin treatment reduced the elevation of MDA and NO levels in the brains of mice relative to the MCAO/R mice. In addition, metformin treatment increased the levels of SOD and GSHPx in serum and brain tissue of MCAO/R mice (Figures 2(a) and 2(d)). Taken together, these data suggest that metformin could reduce oxidative stress in mice with MCAO.

### 3.3. Administration of Metformin Inhibited Oxidative Stress following OGD/R *In Vitro*

**Figure 3:**
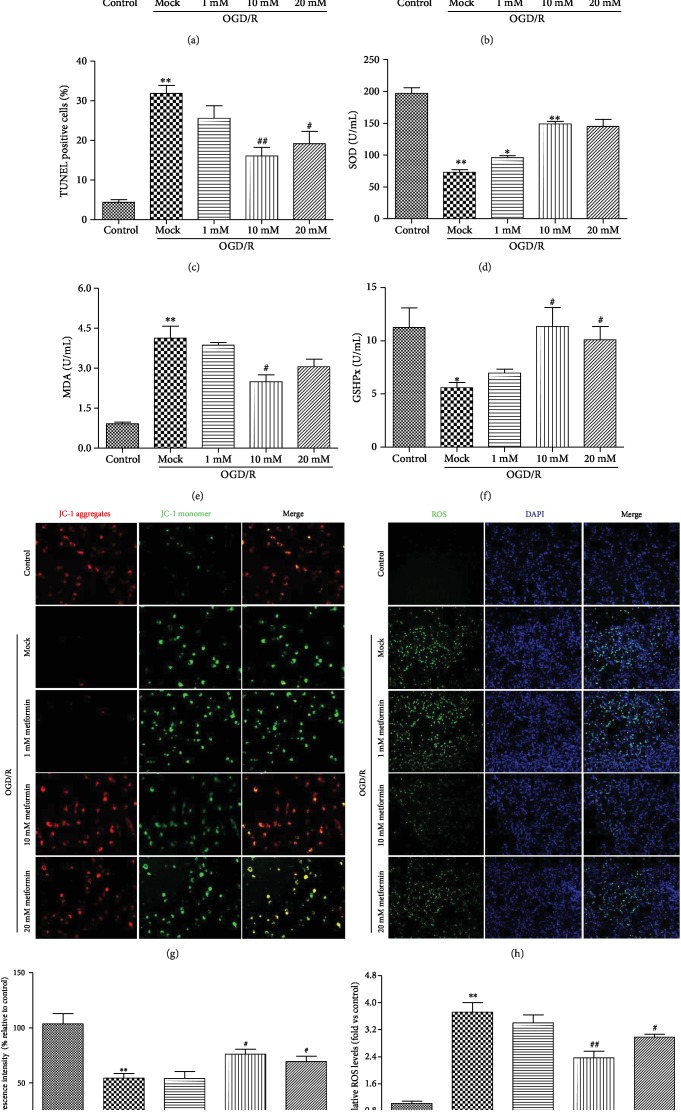
Metformin protected N2a cells against oxidative stress injury under conditions of OGD/R-induced hypoxia. (a) The viability of N2a was determined by CCk8 assay. (b) The levels of LDH were assessed by the LDH kit assay. (c) The apoptosis of N2a was evaluated using TUNEL staining. (d) Activities of SOD in the supernatant of cell culture medium. (e) Levels of MDA in the supernatant of cell culture medium. (f) Activities of GSHPx in the supernatant of cell culture medium. After 24 reperfusion hours, mitochondrial membrane potential (g) and ROS (h) were determined by JC-1 dying or DCFH-DA, respectively. The nucleus was stained with Hoechst 33342 in blue. (i) ROS fluorescence intensity was measured and presented in a bar graph. (j) MMP fluorescence intensity was calculated as the red/green fluorescence ratio. Data are represented as means ± SD (*n* = 3 per group). ^∗^*p* < 0.05 and ^∗∗^*p* < 0.01 versus the control group by 1-way analysis of variance with Tukey's multiple comparison test. ^#^*p* < 0.05 and ^##^*p* < 0.01 versus the mock group by 1-way analysis of variance with Tukey's multiple comparison test. Abbreviations: MMP: mitochondrial membrane potential; ROS: reactive oxygen species; SD: standard deviation. The colored version of the figure is available online.

First, we tested the protective effect of metformin on N2a cell damage induced by OGD/R using cell viability, TUNEL staining, and LDH assays and found that metformin treatment significantly increased cell viability and reduced LDH expression and apoptosis (Figures 3(a)–3(c)). Previous studies demonstrated that OGD/R resulted in significant increases in the levels of NO and MDA and inhibited the activities of SOD and GSHPx in cells subjected to OGD/R compared to the no treatment group. MDA levels as well as the activities of SOD and GSHPx represent the degree of oxidative stress damage to cells. Therefore, we explored the effects of metformin on oxidative stress following OGD/R *in vitro* using MDA, SOD, and GSHPx assays (Figures 3(d)–3(f)). The results show that OGD/R increased NO and MDA levels and decreased SOD and GSHPx levels in N2a cells. Metformin treatment significantly increased the expression of SOD and GSHPx, inhibited the increase in MDA levels, and alleviated the oxidative stress injury induced by OGD/R compared with the model group.

ROS overload and mitochondrial membrane depolarization are the main markers of neuronal apoptosis and are closely related to the stroke process. Therefore, we next determined ROS production and MMP with 2,7-dichlorodihydrogen fluorescein (DCFH-DA) and JC-1 dye, respectively. As can be seen from Figures 3(h) and 3(j), the fluorescence intensity of DCFH-DA was significantly enhanced in N2a cells exposed to OGD/R compared to the control or metformin-treated group. However, metformin treatment significantly reduced ROS production in N2a cells. Similarly, metformin significantly attenuated OGD/R-induced mitochondrial depolarization (Figures 3(g) and 3(i)), suggesting that metformin treatment inhibited OGD/R-induced oxidative stress damage in N2a cells.

### 3.4. Expression of lncRNA-H19, miR-148a-3p, and Rock2 Was Modulated by Metformin following Ischemia Both *In Vivo* and *In Vitro*

**Figure 4:**
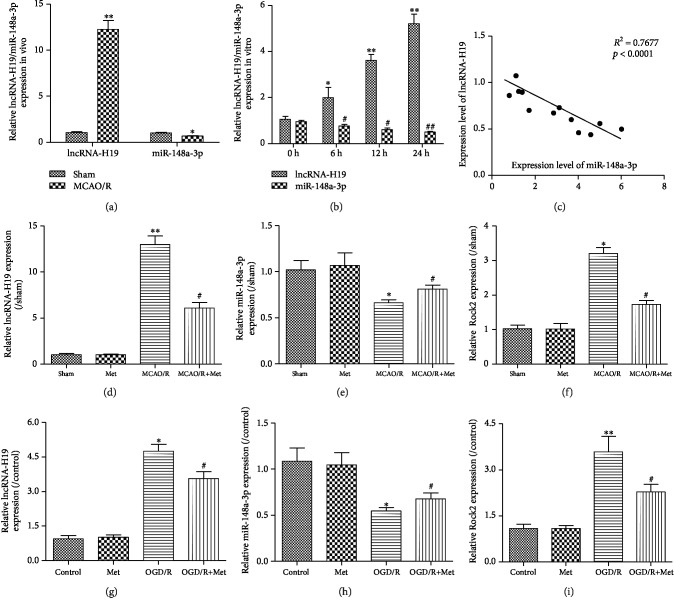
Expression of lncRNA-H19, Rock2, and miR-148a-3p following ischemia both *in vivo* and *in vitro*. (a) RT-PCR assay for the expression of lncRNA-H19 and miR-148a-3p *in vivo*. (b) Expression of lncRNA-H19 and miR-148a-3p at each time point in N2a cells subjected to 4 h OGD and at 0 h, 6 h, 12 h, 24 h, and 48 h of reperfusion (*n* = 3 per group). (c) Pearson's correlation analysis of the relationship between lncRNA-H19 and miR-148a-3p in the brain of mice subjected to MCAO/R surgery. RT-PCR assay for the expression of lncRNA-H19 (d), miR-148a-3p (e), and Rock2 (f) in mice subjected to MCAO/R (*n* = 3 per group). ^∗^*p* < 0.05 and ^∗∗^*p* < 0.01 versus the sham group by 1-way analysis of variance with Tukey's multiple comparison test. ^#^*p* < 0.05 versus the MCAO/R group by 1-way analysis of variance with Tukey's multiple comparison test. RT-PCR was used to detect the expression of lncRNA-H19 (g), miR-148a-3p (h), and Rock2 (i) in N2a cells subjected to OGD/R (*n* = 3 per group). Data are represented as mean ± SD. ^∗^*p* < 0.05 and ^∗∗^*p* < 0.01 versus the control group by 1-way analysis of variance with Tukey's multiple comparison test. ^#^*p* < 0.05 versus the mock group by 1-way analysis of variance with Tukey's multiple comparison test.

As shown in Figures 4(a), 4(d), and 4(f), the expression of lncRNA-H19 and Rock2 in the MCAO/R group was significantly higher than that in the sham group. The level of lncRNA-H19 was also increased in the OGD/R-treated N2a cells compared to the control group (Figure 4(b)). We further discovered that miR-148a-3p expression was significantly lower than the control level in both MCAO/R-treated mice and OGD/R-treated N2a cells (Figures 4(b) and 4(e)). Finally, we found that the expression of lncRNA-H19 was negatively correlated with miR-148a-3p in mouse brain tissue after MCAO/R and negatively correlated with miR-148a-3p in N2a cells exposed to OGD/R (Figures 4(c) and 4(f)). In addition, metformin inhibited the expression of lncRNA-H19 and upregulated the expression of miR-148a-3p *in vitro* and *in vivo* (Figures 4(d)–4(i)).

### 3.5. miR-148a-3p Competitively Binds to lncRNA-H19 and Rock2

**Figure 5:**
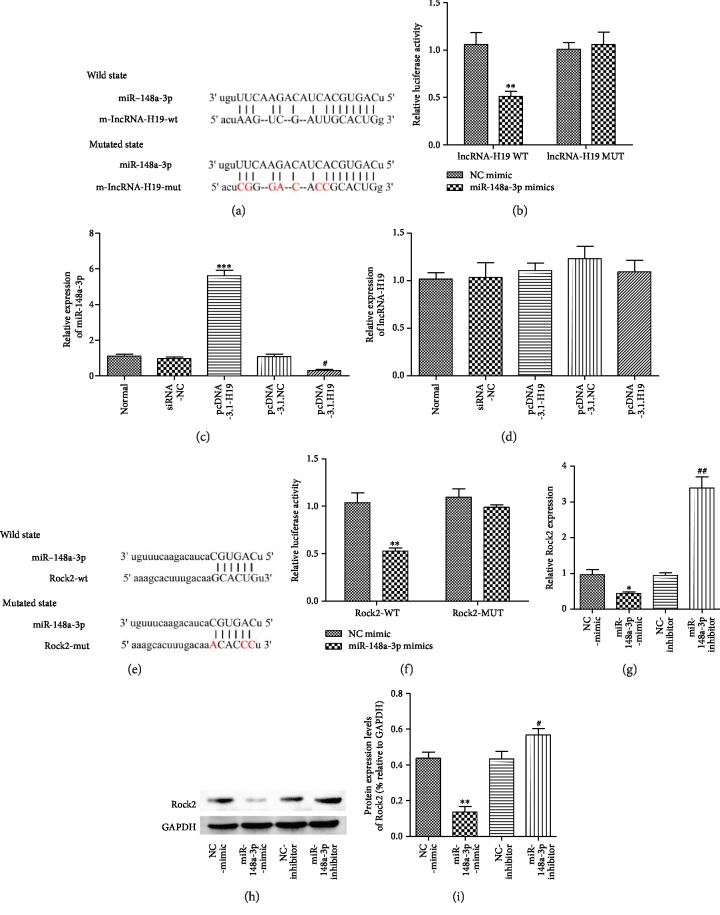
miR-148a-3p competitively binds to lncRNA-H19 and Rock2. (a) The lncRNA-H19 was predicted as a target of miR-148a-3p by online bioinformatic methods (Target Scan and http://microrna.org). (b) The luciferase activity of lncRNA-H19 was declined in N2a cells cotransfected with lncRNA-H19-3′UTR-WT and miR-148a-3p mimic. (c) The expression level of miR-148a-3p was determined after transfection of siRNA-NC, siRNA-H19, pcDNA3.1-NC, and pcDNA3.1-H19. (d) The mRNA expression of lncRNA-H19 was determined in N2a cells cotransfected with NC, NC mimic, miR-148a-3p mimic, NC inhibitor, and miR-148a-3p inhibitor. (e) The miR-148a-3p was predicted as a target of Rock2 by online bioinformatic methods (Target Scan and http://microrna.org). (f) The luciferase activity of Rock2 was declined in N2a cells cotransfected with Rock2-3′UTR-WT and miR-148a-3p mimics. (g–i) The mRNA and protein expression of Rock2 was elevated in N2a cells cotransfected with NC, NC mimic, miR-148a-3p mimic, NC inhibitor, and miR-148a-3p inhibitor. Data are represented as mean ± SD. ^∗^*p* < 0.05 and ^∗∗^*p* < 0.01 versus the NC mimic group by 1-way analysis of variance with Tukey's multiple comparison test. ^#^*p* < 0.05 and ^##^*p* < 0.01 versus the NC inhibitor group by 1-way analysis of variance with Tukey's multiple comparison test.

Bioinformatic analysis using Starbase (http://starbase.sysu.edu.cn) revealed that lncRNA-H19 contains a conserved target site for miR-148a-3p (Figure 5(a)). The dual luciferase reporter assay showed that the miR-148a-3p mimic inhibits the luciferase activity of H19-WT, whereas the negative control (NC) mimic did not. However, the miR-148a-3p mimetic did not change the luciferase activity of H19-MUT (Figure 5(b)). Furthermore, lncRNA-H19 was downregulated or overexpressed in N2a cells after OGD/R, and miR-148a-3p expression was significantly increased or decreased, respectively (Figures 5(c) and 5(d)). These data indicate that lncRNA-H19 may act as a molecular sponge of miR-148a-3p, thereby negatively regulating its effects.

Through online bioinformatic analysis, miR-148a-3p was predicted to directly target the 3′UTR of Rock2 (Figure 5(e)). The luciferase reporter gene assay was used to determine whether the 3′UTR of Rock2 mRNA is a binding target for miR-148a-3p. The results showed that the miR-148a-3p mimetic-driven luciferase activity was significantly reduced compared to the control group, while the Rock2-3′UTR wild-type (WT) group did not change significantly (Figure 5(f)). RT-PCR and western blot showed that the upregulation of miR-148a-3p resulted in a significant decrease in Rock2 mRNA and protein expression compared with the control group. Furthermore, when the miR-148a-3p inhibitor was downregulated using the miR-148a-3p inhibitor, we found the opposite result (Figures 5(g)–5(i)). These results indicate that miR-148a-3p regulates the expression of Rock2 mRNA and protein in N2a cells and that miR-148a-3p directly targets the 3′UTR of Rock2 in N2a cells.

### 3.6. lncRNA-H19 Regulated Rock2 Expression via miR-148a-3p in the OGD/R Model

**Figure 6:**
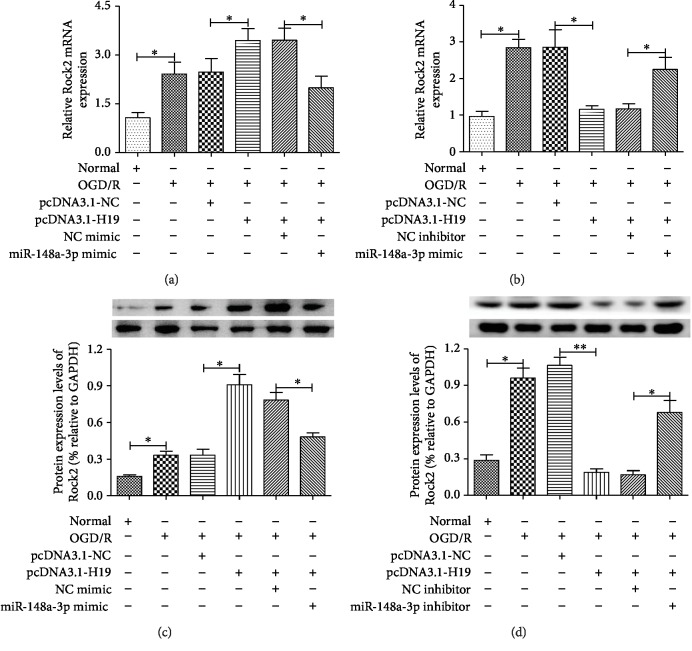
lncRNA-H19 regulates Rock2 expression by miR-148a-3p in OGD/R-induced N2a cells. (a, c) The mRNA and protein expression of Rock2 was decreased in OGD/R-induced N2a cells, and cell transfection of pcDNA3.1-H19 upregulated Rock2 expression in OGD/R-induced N2a cells. However, miR-148a-3p mimic cotransfected into N2a cells reversed the effect of pcDNA3.1-H19 on Rock2 expression. (b, d) The mRNA and protein expression of Rock2 was decreased in N2a cell transfection of siRNA-H19, but miR-148a-3p inhibitor-cotransfected N2a cells reversed the Rock2 expression. Data are represented as mean ± SD; ^∗^*p* < 0.05 and ^∗∗^*p* < 0.01.

Previous results have confirmed that lncRNA-H19 acts as a miR-148a-3p sponge molecule and miR-148a-3p can directly target the 3′UTR of Rock2. So, we speculated that lncRNA-H19 might regulate Rock2 expression by miR-148a-3p in the OGD/R model.  Figure 6(a) shows that Rock2 mRNA levels were significantly decreased in OGD/R-induced N2a cells transfected with pcDNA3.1-H19, whereas Rock2 expression was increased in OGD/R-induced N2a cells transfected with pcDNA3.1-H19. Despite these data, the effect of pcDNA3.1-H19 on Rock2 expression was reversed when the miR-148a-3p mimic was cotransfected with pcDNA3.1-H19 to N2a cells. Next, we executed lncRNA-H19 knockdown by siRNA-H19 to verify whether it could downregulate the expression of Rock2 by miR-148a-3p. As shown in Figure 6(b), the expression of Rock2 was significantly decreased in cells transfected with siRNA-H19, and the cotransfection of the miR-148a-3p inhibitor with siRNA-H19 reversed the decrease in the level of Rock2. In addition, the expression of Rock2 protein also changes with the expression of Rock2 mRNA (Figures 6(c) and 6(d)). These findings reveal that lncRNA-H19 regulates Rock2 expression via miR-148a-3p in the OGD/R model.

### 3.7. Inhibition of lncRNA-H19 Expression Influenced the Effect of Metformin on Oxidative Stress following OGD/R *In Vitro* via Regulation of Rock2/HO-1/Nrf2

**Figure 7:**
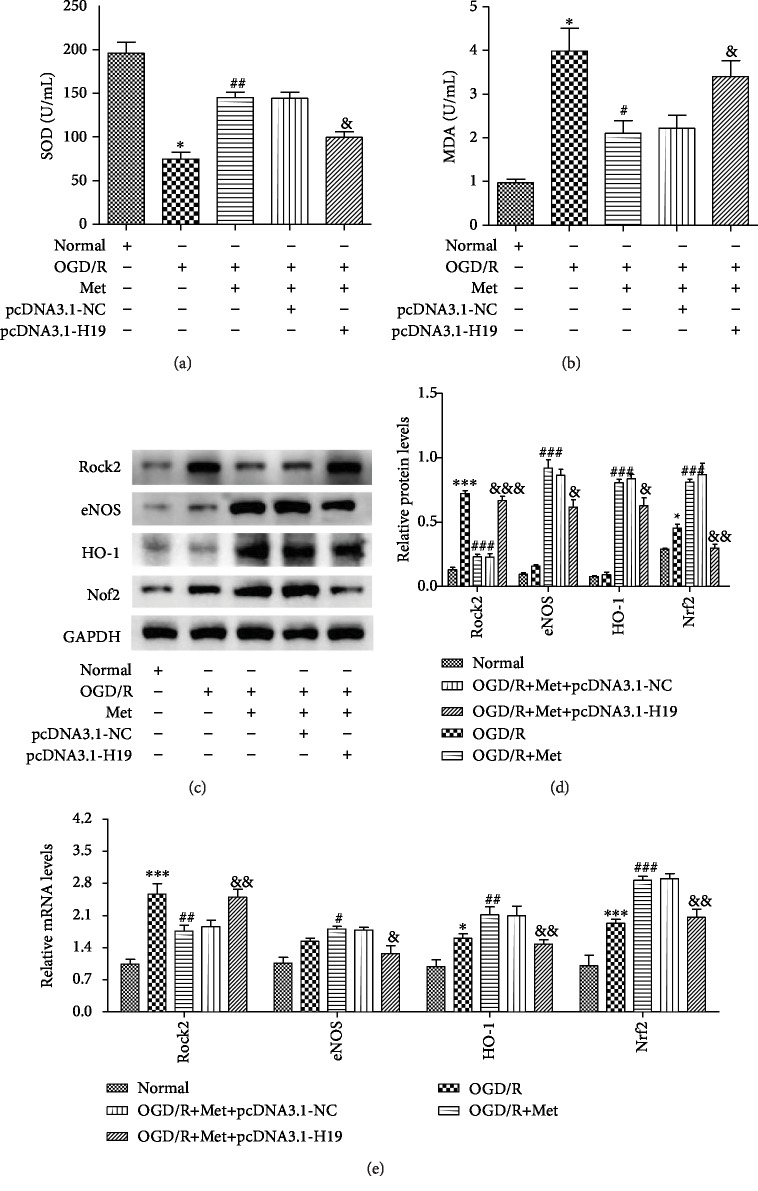
Inhibition of lncRNA-H19 expression influenced the effect of metformin on oxidative stress following OGD/R in vitro via regulation of Rock2/HO-1/Nrf2. (a) Activities of SOD in the supernatant of cell culture medium. (b) Levels of MDA in the supernatant of cell culture medium. (c, d) The protein expressions of Rock2, eNOS, HO-1, and Nrf2 were determined after transfection of pcDNA3.1-NC and pcDNA3.1-H19. (e) The mRNA expressions of Rock2, eNOS, HO-1, and Nrf2 were determined after transfection of pcDNA3.1-NC and pcDNA3.1-H19. Data are represented as mean ± SD. ^∗^*p* < 0.05 and ^∗∗∗^*p* < 0.001 versus the normal group by 1-way analysis of variance with Tukey's multiple comparison test. ^#^*p* < 0.05, ^##^*p* < 0.01, and ^###^*p* < 0.001 versus the OGD/R group by 1-way analysis of variance with Tukey's multiple comparison test. ^&^*p* < 0.05, ^&&^*p* < 0.01, and ^&&&^*p* < 0.001 versus the OGD/R+Met+pcDNA3.1-NC group by 1-way analysis of variance with Tukey's multiple comparison test.

To explore whether lncRNA-H19 was involved in OGD/R-induced oxidative stress, the concentrations of SOD and MDA were assessed in N2a cells cotransfected with pcDNA3.1-H19 and metformin, respectively, using RT-PCR. As shown in Figure 7(a), MDA in the OGD/R group was significantly higher than that in the control group. However, MDA was significantly increased in cells transfected with pcDNA3.1-H19, indicating that pcDNA3.1-H19 aggravated the effect of OGD/R on MDA production. Despite these data, MDA concentrations were significantly reduced when cells were cotransfected with pcDNA3.1-H19 and metformin, suggesting that metformin may reverse the effect of pcDNA3.1-H19 on MDA (Figure 7(b)). The concentration of SOD was opposite to that of MDA. When cells were transfected with pcDNA3.1-H19, the SOD concentration in the OGD/R group increased and the MDA concentration decreased (Figure 7(a)). The level of SOD in N2a cells cotransfected with pcDNA3.1-H19 and metformin also increased, indicating that metformin may also reverse the effect of pcDNA3.1-H19 on SOD production. In addition, we found expected changes in mRNA and protein expression in the oxidative stress gene including eNOS, HO-1, and Nrf2 (Figures 7(c)–7(e)). The changes in the expression of Rock2 and eNOS/HO-1/Nrf2 were consistent with the concentrations of MDA and SOD, respectively, indicating that metformin reversed the effect of pcDNA3.1-H19 on gene expression. These data imply that inhibition of lncRNA-H19 expression influences the effect of metformin on oxidative stress following OGD/R *in vitro* via regulation of Rock2/HO-1/Nrf2.

### 3.8. Metformin Protects against Oxidative Stress Injury Induced by I/R via Regulation of the lncRNA-H19/miR-148a-3p/Rock2 Axis

**Figure 8:**
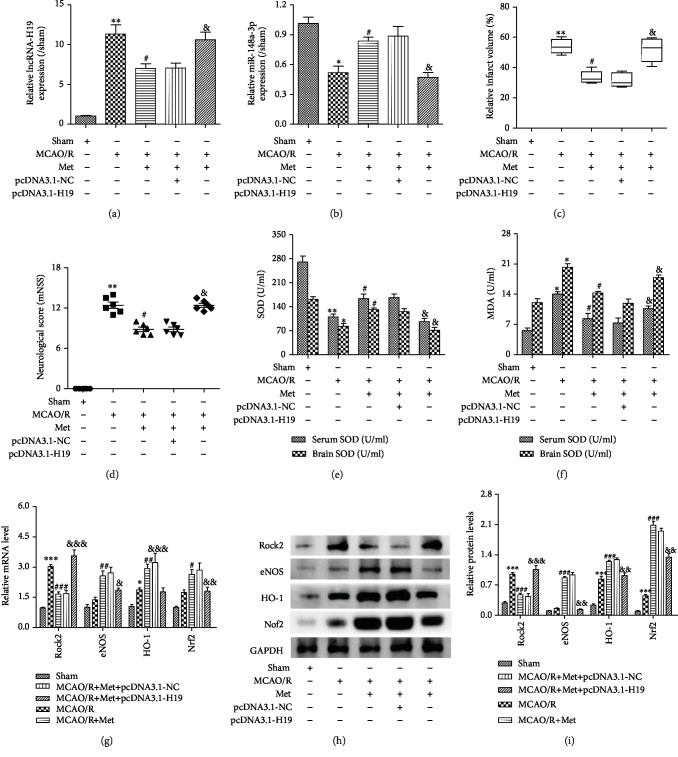
Metformin protects against oxidative stress injury induced by I/R via regulation of the lncRNA-H19/miR-148a-3p/Rock2 axis. (a) The mRNA expressions of lncRNA-H19 were determined after lateral ventricular injection of pcDNA3.1-NC and pcDNA3.1-H19. (b) The level of miR-148a-3p. (c) Statistical analysis of the percentage of infarct volume was determined for each group. (d) Neurological scores after transient middle cerebral artery occlusion (tMCAO) in each groups. (e) Activities of SOD in the serum and the brain. (f) Levels of MDA in the serum and the brain. (g) The mRNA expressions of Rock2, eNOS, HO-1, and Nrf2 were determined after lateral ventricular injection of pcDNA3.1-NC and pcDNA3.1-H19. (h, i) The protein expressions of Rock2, eNOS, HO-1, and Nrf2 were determined after lateral ventricular injection of pcDNA3.1-NC and pcDNA3.1-H19. ^∗^*p* < 0:05, ^∗∗^*p* < 0:01, and ^∗∗∗^*p* < 0:001 versus the sham group by 1-way analysis of variance with Tukey's multiple comparison test. ^#^*p* < 0:05, ^##^*p* < 0:01, and ^###^*p* < 0:01 versus the MCAO/R group by 1-way analysis of variance with Tukey's multiple comparison test. ^&^*p* < 0:05, ^&&^*p* < 0:01, and ^&&&^*p* < 0:001 versus the MCAO/R+Met+NC group by 1-way analysis of variance with Tukey's multiple comparison test.

Our previous results confirmed that metformin can inhibit OGD/R-induced oxidative stress injury via the lncRNA-H19/mir-148a-3p/Rock2 axis. To verify whether the same mechanism is also responsible *in vivo*, we transfected pcDNA3.1-H19 via Lateral ventricular injection in mice and the corresponding indices were observed. Experimental results demonstrated that the high expression of lncRNA-H19 can be reversed by metformin treatment, as shown by the decreases in cerebral infarction volume and neurobehavioral score. Thus, metformin reduces ischemic injury by improving the level of MDA, inhibiting the expression of SOD and at the same time activating the lncRNA-H19/miR-148a-3p/Rock2 axis, inhibiting Nrf2/HO-1 signaling pathway downstream of expression, and decreasing oxidative stress after cerebral ischemia injury. These results confirmed that metformin regulates the lncRNA-H19/miR-148a-3p/Rock2 axis to play a protective role in the brain (Figure 8).

## 4. Discussion

The classic drug design approach is aimed at developing a drug that targets a single protein or signaling pathway for the treatment of disease [37]. However, many neurological diseases such as stroke and neurodegenerative diseases may require multiple treatments due to their complexity [38]. In this study, we found that metformin exerts neuroprotective effects on I/R injury induced by OGD/R or MCAO/R. The mechanism underlying its neuroprotective effects is related to the suppression of oxidative stress and the inhibition of neuronal apoptosis as well as the regulation of the lncRNA-H19/miR-148a-3p/Rock2 axis.

Based on the characteristics of good tolerance, low price, and safety, metformin is widely used to treat type 2 diabetes. Long-term use of metformin is clinically associated with the possibility of reducing stroke. This effect has nothing to do with its hypoglycemic properties [39], suggesting that metformin may represent a therapeutic strategy for ischemic stroke [40]. In both ischemic and hypoxic models, metformin reduces apoptosis in tissues such as muscle, liver, and brain [41, 42]. The use of metformin in brain diseases such as Alzheimer's disease and Huntington's disease has been confirmed [43, 44]. More and more evidence indicates that metformin has neuroprotective effects on stroke [45]. We hypothesized that metformin treatment might represent a potential strategy to prevent stroke. In this study, we used the MCAO/R and OGD/R models to mimic the pathophysiology of ischemic stroke to investigate the neuroprotective effects of metformin *in vivo* and *in vitro*. Administration of metformin significantly improved neurological recovery and alleviated cerebral infarction, pathological changes, and neuronal apoptosis (Figure 1). In the OGD/R model, metformin increased cell activity and reduced apoptosis (Figure 3). These results demonstrated that metformin has neuroprotective effects on cerebral ischemia *in vivo* and *in vitro*.

Oxidative stress is a key factor in cerebral ischemic injury and plays a very important role in this disease [46]. The substantial production of ROS that cannot be eliminated can induce lipid peroxidation, damage membranes of the cell and mitochondria, and induce neuronal apoptosis [47]. Therefore, reducing the damage caused by cerebral ischemia-induced oxidative stress can effectively alleviate the occurrence of ischemic stroke. I/R produces excess NO, ROS, and MOD while downregulating the expression of several endogenous antioxidant enzymes [48, 49], such as SOD, GSHPx, GR, and peroxidase, resulting in oxidative stress in the brain. Among endogenous antioxidant defense mechanisms, SOD can catalyze the incorporation of O^2-^ into H_2_O_2_. GSH is a potent ROS scavenger and its action and recycling requires GSHPx. The enhanced activities of these endogenous antioxidant enzymes confer a degree of protection against oxidative stress. We detected oxidative stress-associated biochemical parameters in the brain and in N2a cells. Metformin could considerably reverse the MCAO/R- or OGD/R-induced decrease in the activities of SOD and GSHPx and the increase in the levels of ROS, MDA, and NO (Figures 2 and 3). These results suggest that metformin could enhance the endogenous antioxidant capacity and subsequently suppress oxidative stress in the brain.

Previous studies have demonstrated that metformin can play a neuroprotective role by inhibiting the oxidative stress response induced by ischemia and hypoxia, so the specific mechanism still needs to be further explored [50]. lncRNA is a type of RNA with a length greater than 200 nt [51]. Instead of coding for protein sequences, it is worth investigating if lncRNAs can affect commensal organisms by regulating miRNAs and related genes and participate in processes such as chromosome remodeling, transcriptional regulation, and RNA degradation [52]. Thus, it has been suggested that lncRNAs could represent a biomarker for stroke. To date, lncRNA-H19, HOTAIR, CCAT1, and MALAT1 have been widely considered to be closely related to the occurrence and development of stroke. Similar to previous studies, this research also showed that upregulation of lncRNA-H19 was accompanied by aggravation of the oxidative stress response (Figure 3), which specifically manifested as inhibition of the increase in SOD and promotion of the release of MDA.

The mechanism by which lncRNAs regulate gene expression is a current hot topic in research, though it presents difficulties [53]. Recent studies have confirmed that lncRNA has many regulatory mechanisms, including almost all aspects of pre- and posttranscriptional processes, including regulation of transcription, protein function, chromatin modification, and posttranscriptional processing [54]. Based on the current background, although lncRNA-H19 seems to continue to promote the development of cerebral ischemia [55], its mechanism of action is still unclear. Using bioinformatic software combined with sequence complementation analysis, this study determined that miR-148a-3p is an inhibitory target of lncRNA-H19. With the gradual deepening of the understanding of RNA, it is not uncommon for a noncoding RNA to be recognized as a target of another noncoding RNA. For example, miR-200, let-7, and miR-675, which are regulated by lncRNA-H19, have been confirmed in previous studies. Importantly, miR-148a-3p not only regulates I/R-induced oxidative stress damage progression, but lncRNA-H19 regulation of oxidative stress progression also requires miR-148a-3p activity. The deletion of miR-148a-3p almost completely reversed the H19 knockdown-induced phenotype. These studies suggest that miR-148a-3p is located downstream of lncRNA-H19 in the signaling cascade that regulates cerebral ischemic processes.

This study indicated that miR-148a-3p could directly target Rock2 to regulate downstream HO-1/Nrf2 signaling, which significantly modulates the oxidative stress process. Nrf2 is a member of the bZIP transcription factor CaMKIIalpha subfamily and plays a very important role in the defense system against oxidative stress [56]. Under steady-state conditions, Nrf2 levels were also maintained at a low level due to proteasome-dependent degradation of Nrf2 and E3 ubiquitin ligase-mediated ubiquitination [57]. High levels of oxidative stress in the liver, heart, kidney, and brain significantly inhibit the degradation of Nrf2 under ischemic stress [58]. Activated Nrf2 can induce the expression of HO-1, indicating that Nrf2 is essential for the regulation of HO-1. Many studies have shown that the HO-1/Nrf2 pathway plays a key role in I/R-induced oxidative stress injury [59, 60]. Furthermore, a previous study demonstrated that a Rock2 inhibitor prevents cell death via activation of the HO-1/Nrf2 signaling in PC12 cells [17]. Thus, the HO-1/NRF2 signaling cascade may be crucial in regulating oxidative stress-induced organ injury. In our current study, metformin inhibited Rock2 by downregulating lncRNA-H19 and then activated HO-1/Nrf2 to inhibit oxidative stress injury induced by cerebral ischemia.

In conclusion, metformin exhibits neuroprotective effects against cerebral ischemia-induced injury though inhibition of oxidative stress and apoptosis. The mechanism underlying this neuroprotective effect involves the regulation of the lncRNA-H19/miR-148a-3p/Rock2 axis and then the activation of the HO-1/Nrf2 pathway. Metformin could be a promising preventive agent for ischemic stroke.

## Figures and Tables

**Table 1:** List of primers used for real-time RT-PCR.

Primer	Symbol	Sequence (5′-3′)
Long-chain noncoding RNA H19	lncRNA-H19	Fwd: GTCAGGACCGTGTTCTCAAGG
		Rev: GCTTCTTTGATGTTACTGAGGGC
Rho-associated protein kinase 2	Rock2	Fwd: TTGGTTCGTCATAAGGCATCAC
		Rev: TGTTGGCAAAGGCCATAATATCT
Endothelial nitric oxide synthase	eNOS	Fwd: GGCTGGGTTTAGGGCTGTG
		Rev: CTGAGGGTGTCGTAGGTGATG
Heme oxygenase-1	HO-1	Fwd: AAGCCGAGAATGCTGAGTTCA
		Rev: GCCGTGTAGATATGGTACAAGGA
Nuclear factor erythroid-2-related factor 2	Nrf2	Fwd: CTGAACTCCTGGACGGGACTA
		Rev: CGGTGGGTCTCCGTAAATGG
MicroRNA 148a-3p	miR-148a-3p	Fwd: AGCAGTTCAGTGCACTACAG
		Rev: GCAGGGTCCGAGGTATTC
U6	U6	Fwd: GCGCGTCGTGAAGCGTTC
		Rev: GTGCAGGGTCCGAGGT
Glyceraldehyde-3-phosphate dehydrogenase	GAPDH	Fwd: CACTCACGGCAAATTCAACGGCA
		Rev: GACTCCACGACATACTCAGCAC

## Data Availability

The data used to support the findings of this study are available from the corresponding authors upon request.
